# Commentary on “A novel concealed posterior axillary approach for scapula glenoid fractures – a multicenter clinical study”

**DOI:** 10.1097/JS9.0000000000004669

**Published:** 2026-01-13

**Authors:** Yuan Cui, Leigang Zheng

**Affiliations:** College of Traditional Chinese Medicine Clinical Medicine, Inner Mongolia Medical University, Hohhot, Inner Mongolia Autonomous Region, China

*Dear Editor*,

We have read with great interest the article by Wang *et al*^[1]^ describing the Tian Yun–Wu Dankai (T-W) posterior axillary approach for Ideberg Ia, II, and selected IV–V glenoid fractures. It should be noted that our article is compliant with the TITAN Guidelines 2025^[2]^. Their multicenter series of 101 patients, reporting high union rates together with favorable functional and cosmetic outcomes, adds important clinical experience to the growing literature on glenoid fracture fixation. In particular, the concealed corridor that avoids rotator cuff detachment and wide posterior dissection is of clear practical relevance.

Pattern selection is, as the authors emphasize, central to the safe application of the T-W approach^[1]^. It would be helpful to better understand how surgeons approached “borderline” fracture configurations, especially comminuted Ideberg II or IV–V injuries extending beyond the 1:00–7:00 arc. Earlier work by Armitage *et al* and Ideberg *et al*^[3,4]^ has underlined the considerable variability in glenoid fracture morphology. In future prospective cohorts, a preoperative CT-based decision scheme or flowchart outlining when a combined or alternative approach is preferred could further support consistent use of this technique across centers.

The low rate of postoperative sensory disturbance and absence of major neurovascular complications in this series are also reassuring, given the proximity of the axillary nerve and circumflex scapular vessels^[1]^. Nonetheless, anatomical and clinical studies indicate that even limited posterior exposure may occasionally be associated with axillary neuropraxia or subscapularis dysfunction, particularly when muscle detachment is required^[5,6]^. Stratifying functional scores and range of motion by fracture pattern and by the presence or absence of perioperative nerve symptoms in future reports could help readers appreciate the practical “safety margin” of the T-W window in everyday practice.

In summary, Wang *et al*^[1]^ are to be congratulated for presenting an elegant posterior axillary approach for challenging glenoid fractures. Their experience suggests that the T-W corridor may become a useful complement to established anterior and posterior techniques, particularly in patients for whom scar placement is an important consideration. Larger comparative studies with standardized rehabilitation protocols and longer radiographic follow-up will be valuable in defining the precise role of this approach within the contemporary management of glenoid fractures.

## Data Availability

No new data were generated.
